# Supplementary material for: Effectiveness of care coordination interventions delivered to stroke survivors in low and middle-income countries: Systematic review and meta-analysis protocol
Source: PLoS One. 2025 May 16;20(5):e0324040. doi: 10.1371/journal.pone.0324040 (PMC12083812; doi:10.1371/journal.pone.0324040)
Supplement: S2 Table — (PDF) [file pone.0324040.s003.pdf]

Data Extraction Form adapted from the Cochrane Collaboration

Title of the systematic review: Effectiveness of Stroke Coordinated Care Interventions Delivered to Stroke Survivors in LMICs: Systematic Review and Meta-Analysis

Protocol Registration no: CRD42024587311

This form has been developed by adopting and customizing the "Data collection form for intervention review – RCTs and non-RCTs" of The Cochrane Collaboration.

Part A: For Interventional Studies Only

General Information

**Study ID**<br>*(e.g. author name, year)*	
**Country(ies) of study**	
**Form completed by**	
**Study author contact details**	
**Setting**<br>(e.g., hospital, community, rural, urban)	
**Year data was collected**	
**Notes:**	

Characteristics of included studies

Methods

	**Descriptions as stated in report/paper**	**Location in text or source** *(pg & ¶/fig/table/other)*
**Randomization method** (if applicable e.g. simple, block, stratified)		
**Design** *(indicate whether the study is a randomized controlled trial, a non-randomized control trial or a pre-test post-test)*		

**Blinding** (if applicable e.g. single, double or open-label)		
**Unit of allocation** *(by individuals, cluster/ groups or body parts)*		
**Start date/year of study**		
**End date/year of study**		
**Total study duration** (*in weeks*)		
**Notes:**		

Participants

	**Description** *Include comparative information for each intervention or comparison group if available*	**Location in text or source** *(pg & ¶/fig/table/other)*
**Type of stroke** (ischemic, hemorrhagic, or both)		
**Stroke sub-type**		
**Stroke severity***(report average NIHSS score, other severity scale score)		
**Setting** *(including health setting or community, type of clinical setting)*		
**Inclusion criteria** (provide a bullet point list of inclusion criteria)		
**Exclusion criteria** (provide a bullet point list of exclusion criteria)		
**Method of recruitment of participants** *(e.g. phone, mail, clinic patients, voluntary)*		
**Total no. randomized**		
**Total sample size**		

**Clusters** *(if applicable, no., type, no. people per cluster)*			
**No. randomised per group** *(specify whether no. people or clusters)*	*Group 1 name*	*Group 2 name*	
**No. per group** *(specify whether no. people or clusters)*	*Group 1 name*	*Group 2 name*	
**No. missing** *(if overall, e.g. exclusions & withdrawals, whether or not missing from analysis)*			
**Reasons missing**			
**No. missing** *(if by group, e.g. exclusions & withdrawals, whether or not missing from analysis)*			
**Reasons missing**			
**No. participants moved from one group to another**			
**Reasons moved**			
**Baseline imbalances**			
**Age** (indicate the mean/median age of the various groups, pre-post groups involved in the study)	*Group 1 name*		
**Sex** (indicate the proportion of women in the various groups, pre-post groups involved in the study)			
**Stroke severity** (indicate the mean/median NIHSS or other method used for various groups, pre-post groups involved in the study)			
**Stroke type**(if applicable indicate the proportion of hemorrhagic stroke for various groups, pre-post groups involved in the study)			

**Functional status** (indicate the mean/median functional status (e.g., using the Modified Rankin Scale, Barthel Index) or other method used for various groups, pre-post groups involved in the study. If multiple scales are used, list each one)		
**Cognitive status** (indicate the mean/median functional status (e.g., using the MINI, Montreal Cognitive Assessment) or other method used for various groups, pre-post groups involved in the study. If multiple scales are used, list each one)		
**Comorbidities** (indicate all comorbid conditions and their various proportions for the various groups at baseline)		
**Educational status** (indicate all levels of education and their various proportions for the various groups at baseline)		
**Income/socio-economic status** (indicate all levels of income/socio-economic status and their various proportions for the various groups at baseline)		
**Other relevant sociodemographic**		
**Notes:**		

Intervention groups

Copy and paste table for each intervention and comparison group

Intervention Group 1

	Description as stated in report/paper	Location in text or source *(pg & ¶/fig/table/other)*
**Group name** *(from paper or abbreviation)*		
**Theoretical basis** *(include key references)*		
**Number randomized to intervention**		
**Timing (duration)**		
**Description of care coordination intervention** (Provide a detailed description of the care coordination intervention, ensuring it includes key components of multiple participants' involvement, information exchange, and clear roles. If these elements are not adequately addressed, mark "does not meet care coordination definition.)		
**Components of care coordination intervention**		
**Multiple participants** **(***specify the multiple health professionals involved in the intervention and their roles)*		
**Information exchange between participants** (extract details on how information was exchanged between participants e.g interprofessional meetings, phone call/meetings, electronic health records exchange etc.)		
**Understanding roles** (report how participants familirise themselves with individual roles or attempted to understand roles of others eg. Trainings that clarified roes, communication of responsibilities etc).		
**Activities of care coordination**		

**Assessing patient needs (**specify methods used to assess patient needs (e.g. surveys, interviews, assessment) and indicate who conducted the assessment and key needs identified)		
**Developing care plans** (Describe the process of creating care plans, including who was involved (e.g., patient, family, healthcare team). Note if the care plans were individualized and any specific goals set within them.)		
**Providing direct care** (Record the types of direct care services provided (e.g., medication administration, physical therapy). Specify who delivered the care and the context (e.g., in-home, outpatient)		
**Monitoring responses to care** (Document the strategies used for monitoring patient responses (e.g., follow-up visits, phone calls). Indicate the frequency of monitoring and any specific outcomes assessed.)		
**Partnering with community resources** (Identify community resources involved and the nature of partnerships established (e.g., referrals to social services, support groups). Document how patients were connected to these resources.)		
**Fostering collaboration within MDT (**Describe the activities that promoted collaboration among MDT members (e.g., team meetings, shared decision-making). Specify how often these collaborations occurred.)		

**Facilitating care transitions** (Record the processes used to facilitate care transitions (e.g., discharge planning, transfer protocols). Indicate which team members were involved in these transitions.)		
**Negotiating responsibilities** (Record instances where responsibilities were discussed or negotiated among team members. Include who was involved in these discussions and any agreements made.)		
**Establishing shared responsibilities (**Document activities that defined shared responsibilities among team members (e.g., joint goals, collaborative care plans). Note how these responsibilities were communicated to the team.)		
**Exercising leadership** (Describe any leadership activities that supported care coordination (e.g., designated team leader roles, leadership training). Specify how leadership was enacted and its impact on coordination.)		
**Duration of treatment period** (Document the overall duration of the treatment period for the intervention (e.g., total weeks/months)		
**Timing** *(Specify the frequency of care coordination activities (e.g., daily, weekly) and the duration of each episode (e.g., minutes per session).*		

**Delivery** *(Describe the type of delivery methods used (e.g., in-person, telehealth), the medium (e.g., phone, video), intensity (e.g., high, moderate), and fidelity (e.g., adherence to the intervention protocol).*		
**Co-interventions** *(Document any additional interventions that occurred simultaneously with the care coordination intervention (e.g., medication management, physical therapy). Specify the nature of these co-interventions, how they were implemented, and their intended outcomes.)*		
**Notes:**		

Data and analysis

Copy and paste the appropriate table for each outcome, as required.

Dichotomous outcome

**Outcome definition** *(Clearly define each outcome being measured. If relevant, include diagnostic criteria used for the outcome (e.g., clinical guidelines, cut-off values)*		
**Name of measuring tool**		
**Cut off point**		
**Person measuring/reporting** (Identify the person or group responsible for measuring and/or reporting the outcome (e.g., a clinician, nurse, patient self-report)		

**Unit of measurement** *(Specify the unit used to measure the outcome (e.g., mg/dL, mmHg, scale points). If units are not provided or inappropriate, mark "not reported.)*					
**Scales: upper and lower limits** *(indicate whether high or low score is good)*					
**Is outcome/tool validated?**					
**Imputation of missing data** *(Document the method used for handling missing data (e.g., multiple imputation, last observation carried forward). Indicate if assumptions were made for intent-to-treat (ITT) analysis, and describe them if relevant.)*					
**Results**	**Intervention**				**Comparison**
Subgroup *(indicate the subgroup, copy rows as needed)*					
**Results (**Indicate time point of measurement and copy row as needed)					
**Change from baseline**					
**Time points measured but not reported**					
**Any other results reported** *(e.g. odds ratio, risk difference, CI or P value)*					
**Unit of analysis** *(by individuals, cluster/groups or body parts)*					

Continuous outcome

	**Description as stated in report/paper**	**Location in text or source** *(pg & ¶/fig/table/other)*

**Outcome definition** *(Clearly define each outcome being measured. If relevant, include diagnostic criteria used for the outcome (e.g., clinical guidelines, cut-off values)*			
**Person measuring/reporting** (Identify the person or group responsible for measuring and/or reporting the outcome (e.g., a clinician, nurse, patient self-report)			
**Unit of measurement** *(Specify the unit used to measure the outcome (e.g., mg/dL, mmHg, scale points). If units are not provided or inappropriate, mark "not reported.)*			
**Scales: upper and lower limits** *(indicate whether high or low score is good)*			
**Is outcome/tool validated?**	☐ Yes ☐ No ☐ Unclear		
**Minimally important difference** (Extract the minimally important difference (MID) if reported, defined as the smallest change in the outcome that is clinically meaningful to patients or providers. If no MID is provided, indicate "not reported.)			
**Imputation of missing data** *(Document the method used for handling missing data (e.g., multiple imputation, last observation carried forward). Indicate if assumptions were made for intent-to-treat (ITT) analysis, and describe them if relevant.)*			
**Baseline results of the outcome**			
**Results**	**Intervention**	**Comparison**	

	Mean	SD *(or other variance, specify)*	No. participants	Mean	SD *(or other variance, specify)*	No. participants	
(time point or subgroup, copy rows as needed)							
**Change from Baseline**							
**Results** (Indicate time point of measurement and copy row as needed)	Mean	SD *(or other variance, specify)*	Mean	No. participants			
**Change from baseline**							
**Time points measured but not reported**							
**Any other results reported** *(e.g. mean difference, CI, P value)*							
**Unit of analysis** *(by individuals, cluster/groups or body parts)*							
**Reanalysis possible?**	☐ Yes ☐ No ☐ Unclear						
**Reanalysed results**							
**Notes:**							

Other information

	Description as stated in report/paper	Location in text or source *(pg & ¶/fig/table/other)*
**Key conclusions of study authors**		
**References to other relevant studies**		
**Correspondence required for further study information** *(from whom, what and when)*		

**Notes:**

Part B: For Observational Studies Only

**Study ID**<br>*(e.g. author name, year)*	
**Country(ies) of study**	
**Form completed by**	
**Study author contact details**	
**Setting**<br>(e.g., hospital, community,<br>rural, urban)	

Year data was collected
**Notes:**

Characteristics of included studies

Methods

	Descriptions as stated in report/paper	Location in text or source *(pg & ¶/fig/table/other)*
**Design** *(Specify the observational study design used (e.g., cohort, case-control, cross-sectional)*		
**Sampling method** (Record the method used to select participants (e.g., random sampling, convenience sampling).		
**Start date/year of study**		
**End date/year of study**		
**Total study duration** (*in weeks*)		
**Notes:**		

Participants

	**Description** *Include comparative information for each intervention or comparison group if available*	Location in text or source *(pg & ¶/fig/table/other)*
**Type of stroke** (ischemic, hemorrhagic, or both)		
**Stroke sub-type**		
**Stroke severity***(report average NIHSS score, other severity scale score)		
**Setting** *(including health setting or community, type of clinical setting)*		

**Inclusion criteria** (provide a bullet point list of inclusion criteria)			
**Exclusion criteria** (provide a bullet point list of exclusion criteria)			
**Method of recruitment of participants** *(e.g. phone, mail, clinic patients, voluntary)*			
**Total sample size**			
**No. per group** *(specify whether no. people or clusters)*	*Group 1 name*	*Group 2 name*	
**No. missing** *(if overall, e.g. exclusions & withdrawals, whether or not missing from analysis)*			
**Reasons missing**			
**No. missing** *(if by group, e.g. exclusions & withdrawals, whether or not missing from analysis)*			
**Reasons missing**			
**No. participants moved from one group to another**			
**Reasons moved**			
**Baseline imbalances**			
**Age** (indicate the mean/median age of the various groups, pre-post groups involved in the study)	*Group 1 name*		
**Sex** (indicate the proportion of women in the various groups, pre-post groups involved in the study)			
**Stroke severity** (indicate the mean/median NIHSS or other method used for various groups, pre-post groups involved in the study)			

**Stroke type**(if applicable indicate the proportion of hemorrhagic stroke for various groups, pre-post groups involved in the study)		
**Functional status** (indicate the mean/median functional status (e.g., using the Modified Rankin Scale, Barthel Index) or other method used for various groups, pre-post groups involved in the study. If multiple scales are used, list each one)		
**Cognitive status** (indicate the mean/median functional status (e.g., using the MINI, Montreal Cognitive Assessment) or other method used for various groups, pre-post groups involved in the study. If multiple scales are used, list each one)		
**Comorbidities** (indicate all comorbid conditions and their various proportions for the various groups at baseline)		
**Educational status** (indicate all levels of education and their various proportions for the various groups at baseline)		
**Income/socio-economic status** (indicate all levels of income/socio-economic status and their various proportions for the various groups at baseline)		
**Other relevant sociodemographics**		

**Attrition** (Document the number of participants lost to follow-up or who dropped out of the study. If reasons for attrition are provided, extract them as well.)		
**Notes:**		

Exposure groups

Copy and paste table for each intervention and comparison group

Care coordination exposure group

	**Description as stated in report/paper**	**Location in text or source** *(pg & ¶/fig/table/other)*
**Group name** *(from paper or abbreviation)*		
**Number in the group**		
**Timing (duration)** (Record the length of time participants were exposed to the care coordination intervention (e.g., number of weeks or months the intervention was delivered). If different exposure periods exist between groups, document each group's exposure duration separately)		

**Description of care coordination intervention** (Provide a detailed description of the exposure or intervention (e.g., care coordination activities, services received). Include any specific details about what constituted the exposure, how it was delivered, and the nature of care coordination involved. If more than one exposure group exists (e.g., varying intensities of care coordination), describe each group separately)		
**Components of care coordination exposure**		
**Multiple participants** **(***specify the multiple health professionals involved in the intervention and their roles)*		
**Information exchange between participants** (extract details on how information was exchanged between participants e.g interprofessional meetings, phone call/meetings, electronic health records exchange etc.)		
**Understanding roles** (report how participants familirise themselves with individual roles or attempted to understand roles of others eg. Trainings that clarified roes, communication of responsibilities etc).		
**Activities of care coordination**		
**Assessing patient needs** **(**specify methods used to assess patient needs (e.g. surveys, interviews, assessment) and indicate who conducted the assessment and key needs identified)		

**Developing care plans** (Describe the process of creating care plans, including who was involved (e.g., patient, family, healthcare team). Note if the care plans were individualized and any specific goals set within them.)		
**Providing direct care** (Record the types of direct care services provided (e.g., medication administration, physical therapy). Specify who delivered the care and the context (e.g., in-home, outpatient)		
**Monitoring responses to care** (Document the strategies used for monitoring patient responses (e.g., follow-up visits, phone calls). Indicate the frequency of monitoring and any specific outcomes assessed.)		
**Partnering with community resources** (Identify community resources involved and the nature of partnerships established (e.g., referrals to social services, support groups). Document how patients were connected to these resources.)		
**Fostering collaboration within MDT (**Describe the activities that promoted collaboration among MDT members (e.g., team meetings, shared decision-making). Specify how often these collaborations occurred.)		
**Facilitating care transitions** (Record the processes used to facilitate care transitions (e.g., discharge planning, transfer protocols). Indicate which team members were involved in these transitions.)		

**Negotiating responsibilities** (Record instances where responsibilities were discussed or negotiated among team members. Include who was involved in these discussions and any agreements made.)		
**Establishing shared responsibilities (**Document activities that defined shared responsibilities among team members (e.g., joint goals, collaborative care plans). Note how these responsibilities were communicated to the team.)		
**Exercising leadership** (Describe any leadership activities that supported care coordination (e.g., designated team leader roles, leadership training). Specify how leadership was enacted and its impact on coordination.)		
**Duration of treatment period** (Document the overall duration of the treatment period for the intervention (e.g., total weeks/months)		
**Timing** *(Specify the frequency of care coordination activities (e.g., daily, weekly) and the duration of each episode (e.g., minutes per session).*		
**Delivery** *(Describe the type of delivery methods used (e.g., in-person, telehealth), the medium (e.g., phone, video), intensity (e.g., high, moderate), and fidelity (e.g., adherence to the intervention protocol).*		

**Co-exposures** *(Document any additional exposures participants may have received during the study period, including treatments or interventions outside the care coordination activities. If co-exposures were discussed in the study (e.g., medications, other therapies), extract details. If not reported, indicate "not reported.)*		
**Notes:**		

Copy and paste table for each intervention and comparison group

Other exposure group

	**Description as stated in report/paper**	**Location in text or source** *(pg & ¶/fig/table/other)*
**Group name** *(Extract the definition of the provided in the study)*		
**Number in the group**		
**Timing (duration)** (Record the length of time participants were exposed (e.g., number of weeks or months the intervention was delivered). If different exposure periods exist between groups, document each group’s exposure duration separately)		

**Description of exposure** (Provide a detailed description of the exposure or intervention (e.g., activities, services received). Include any specific details about what constituted the exposure, how it was delivered, and the nature of care coordination involved. If more than one exposure group exists (e.g., varying intensities of care coordination), describe each group separately)		
**Duration of treatment period** (Document the overall duration of the treatment period for the intervention (e.g., total weeks/months)		
**Timing** *(Specify the frequency of care coordination activities (e.g., daily, weekly) and the duration of each episode (e.g., minutes per session).*		
**Delivery** *(Describe the type of delivery methods used (e.g., in-person, telehealth), the medium (e.g., phone, video), intensity (e.g., high, moderate), and fidelity (e.g., adherence to the intervention protocol).*		
**Co-exposures (***Document any additional exposures participants may have received during the study period, including treatments or interventions outside the care coordination activities. If co-exposures were discussed in the study (e.g., medications, other therapies), extract details. If not reported, indicate "not reported.)*		
**Notes:**		

Data and analysis

Copy and paste the appropriate table for each outcome, as required.

Dichotomous outcome

**Outcome definition** *(Record the definition of the dichotomous outcome, including diagnostic criteria if relevant (e.g., mortality, stroke recurrence).*			
**Name of measuring tool**			
**Cut off point**			
**Person measuring/reporting** (Identify the person or group responsible for measuring and/or reporting the outcome (e.g., a clinician, nurse, patient self-report)			
**Definition of 'success' or 'failure'** (Document how the study defined success or failure in relation to the dichotomous outcome (e.g., survival, non-recurrence of stroke).			
**Method of ascertainment** (Record the method used to ascertain the outcome (e.g., medical records, direct observation, patient-reported).			
**Unit of measurement** *(Specify the unit of measurement used for the dichotomous outcome (e.g., number of events, proportion of participants).*			
**Scales: upper and lower limits** *(indicate whether high or low score is good)*			
**Is outcome/tool validated?**			

**Imputation of missing data** *(Document the method used for handling missing data (e.g., multiple imputation, last observation carried forward). Indicate if assumptions were made for intent-to-treat (ITT) analysis, and describe them if relevant.)*					
**Outcome timing** (Extract the time points at which the dichotomous outcome was measured (e.g., at discharge, 30 days, 6 months). If multiple time points were used, record each one)					
**Results** (Indicate time point of measurement and copy row as needed)	**No. Exposed to care coordination**	No. with event	**No. Exposed to other groups**	No. with event	**No exposed to other group**
Subgroup *(indicate the subgroup, copy rows as needed)*					
**Time points measured but not reported**					
**Any other results reported** *(e.g. odds ratio, risk difference, risk ratio, CI or P value)*					
**Adjustment for confounders** (: Document whether the analysis of dichotomous outcomes was adjusted for confounding variables (e.g., age, sex, baseline health conditions). If adjustments were made, extract the list of confounders)					

Continuous outcome

	Description as stated in report/paper		Location in text or source *(pg & ¶/fig/table/other)*
**Outcome definition** *(Clearly define the continuous outcome being measured (e.g., blood pressure, cholesterol level, stroke severity score). Specify any diagnostic criteria or cut-offs, if relevant. If the outcome is not clearly defined, indicate "not reported.")*			
**Person measuring/reporting** (Identify the person or group responsible for measuring and/or reporting the outcome (e.g., a clinician, nurse, patient self-report)			
**Unit of measurement** *(Specify the unit used to measure the outcome (e.g., mg/dL, mmHg, scale points). If units are not provided or inappropriate, mark "not reported.)*			
**Scales: upper and lower limits** *(indicate whether high or low score is good)*			
**Is outcome/tool validated?**	☐ Yes ☐ No ☐ Unclear		
**Minimally important difference** (Extract the minimally important difference (MID) if reported, defined as the smallest change in the outcome that is clinically meaningful to patients or providers. If no MID is provided, indicate "not reported.)			

**Imputation of missing data** *(Document the method used for handling missing data (e.g., multiple imputation, last observation carried forward). Indicate if assumptions were made for intent-to-treat (ITT) analysis, and describe them if relevant.)*							
**Baseline results of the outcome**							
**Results**	**Exposure group (care coordination)**			**Exposure group**			
	Mean	SD *(or other variance, specify)*	No. participants	Mean	SD *(or other variance, specify)*	No. participants	
(time point or subgroup, copy rows as needed)							
**Change from Baseline**							
**Results** (Indicate time point of measurement and copy row as needed)	Mean	SD *(or other variance, specify)*	Mean	SD *(or other variance, specify*			
**Time points measured but not reported**							
**Any other results reported** *(e.g. mean difference, CI, P value)*							
**Unit of analysis** *(by individuals, cluster/groups or body parts)*							
**Reanalysis possible?**	☐ Yes ☐ No ☐ Unclear						
**Reanalysed results**							
**Notes:**							

Other information

	**Description as stated in report/paper**	**Location in text or source** *(pg & ¶/fig/table/other)*

**Key conclusions of study authors**		
**References to other relevant studies**		
**Correspondence required for further study information** *(from whom, what and when)*		
**Notes:**		
